# Artificial intelligence (AI) diagnostic tools: utilizing a convolutional neural network (CNN) to assess periodontal bone level radiographically—a retrospective study

**DOI:** 10.1186/s12903-022-02436-3

**Published:** 2022-09-13

**Authors:** Ghala Alotaibi, Mohammed Awawdeh, Fathima Fazrina Farook, Mohamed Aljohani, Razan Mohamed Aldhafiri, Mohamed Aldhoayan

**Affiliations:** 1 College of Dentistry, King Saud bin Abdulaziz University for Health Sciences, Riyadh, Saudi Arabia; 2 King Abdullah International Medical Research Centre, Riyadh, Saudi Arabia; 3 Department of Health Informatics, King Saud bin Abdulaziz University for Health Sciences, Riyadh, Saudi Arabia; 4 Department of Oral Maxillofacial Radiology, King Saud University, Riyadh, Saudi Arabia

**Keywords:** CNN, Artificial intelligence, Teeth, Bone level, Periodontitis, Learning machine, VGG-16

## Abstract

**Background:**

The purpose of this investigation was to develop a computer-assisted detection system based on a deep convolutional neural network (CNN) algorithm and to evaluate the accuracy and usefulness of this system for the detection of alveolar bone loss in periapical radiographs in the anterior region of the dental arches. We also aimed to evaluate the usefulness of the system in categorizing the severity of bone loss due to periodontal disease.

**Method:**

A data set of 1724 intraoral periapical images of upper and lower anterior teeth in 1610 adult patients were retrieved from the ROMEXIS software management system at King Saud bin Abdulaziz University for Health Sciences. Using a combination of pre-trained deep CNN architecture and a self-trained network, the radiographic images were used to determine the optimal CNN algorithm. The diagnostic and predictive accuracy, precision, confusion matrix, recall, F1-score, Matthews Correlation Coefficient (MCC), Cohen Kappa, were calculated using the deep CNN algorithm in Python.

**Results:**

The periapical radiograph dataset was divided randomly into 70% training, 20% validation, and 10% testing datasets. With the deep learning algorithm, the diagnostic accuracy for classifying normal versus disease was 73.0%, and 59% for the classification of the levels of severity of the bone loss. The Model showed a significant difference in the confusion matrix, accuracy, precision, recall, f1-score, MCC and Matthews Correlation Coefficient (MCC), Cohen Kappa, and receiver operating characteristic (ROC), between both the binary and multi-classification models.

**Conclusion:**

This study revealed that the deep CNN algorithm (VGG-16) was useful to detect alveolar bone loss in periapical radiographs, and has a satisfactory ability to detect the severity of bone loss in teeth. The results suggest that machines can perform better based on the level classification and the captured characteristics of the image diagnosis. With additional optimization of the periodontal dataset, it is expected that a computer-aided detection system can become an effective and efficient procedure for aiding in the detection and staging of periodontal disease.

## Background

Periodontitis (PD), a multifactorial and complex inflammatory disease in tooth-supporting tissues, is categorized by the loss of periodontal tissue support [5]. It is considered the second most prevalent oral disease globally (20–50%) and is the primary cause of tooth loss in adults [19]. Though the microbial plaque biofilm initiates the process, progression is largely due to an exaggerated host immune-inflammatory response [5]. It is a major public health problem with a significant impact on an individual’s quality of life [19].

Despite the latest advances in treatment modalities, there has not been a significant improvement in the methodology for detecting alveolar bone loss and assessing the severity of the bone loss in the compromised teeth. Radiographs, such as panoramic/periapical and bitewing radiographs as well as periodontal probing, are widely used as objective diagnostic tools for diagnosing and predicting periodontally compromised teeth (PCT). Clinical diagnostic and prognostic judgment depends greatly on empirical evidence [20].

Artificial intelligence (AI) has primarily been used in dentistry to improve the accuracy and efficiency of diagnosis, which is critical to achieving the best outcomes for procedures, and provide superior patient care [8]. AI approaches may be beneficial because they provide a more effective diagnostic process when combined with clinical assessment. Using image recognition, classification, and segmentation, AI may enhance dental efficiency. Convolutional neural networks (CNNs), the latest core model of artificial neural networks, and deep learning in computer vision include image recognition and segmentation, which can be used as a supplement to radiographs to detect periodontal disease. CNNs can detect edges and capture patterns in PCT images. Through their multiple convolutional and hidden layers, deep CNN algorithms can learn hierarchical feature representations and capture regional patterns from the PCT images.

To date, there have only been a few studies that investigated the use of deep learning in the diagnosis of PCT. CNN-based methods were proposed for detecting radiographic bone loss (RBL) on dental panoramic radiographs [2–11, 16–22, 25]. Studies have also evaluated deep CNN for determining peri-implant marginal bone loss on dental periapical radiographs [6]. The CNN was used in the detection of periodontally compromised posterior teeth on intraoral radiographs [15].

The purpose of the current study was to evaluate the potential usefulness and accuracy of deep CNN algorithms for detecting an alveolar bone loss in incisor teeth in periapical radiographs and the severity of the bone loss in the periodontally compromised incisor teeth.

## Methods

The study was conducted in the College of Dentistry, King Saud Bin Abdulaziz University for Health Sciences, and approved by the Institutional Review Board of King Abdullah International Medical Research Center at Riyadh, Saudi Arabia (SP20-234-R).

A data set of 1724 intraoral periapical images of upper and lower anterior teeth from randomly selected periodontitis adult patients between 2015 and 2020 was retrieved from the ROMEXIS 6.0 software (Planmeca, Finland). Periapical images of patients aged 12 years or younger, as well as images with severe noise or haziness or showing teeth that were partially present or severely distorted, had undergone apical surgery with root resection, with a full restorative crown, or teeth with a shape that deviated from normal anatomical structures, were excluded.

All periapical images were annotated and examined by three independent examiners, including a periodontist who collected, deciphered, and categorized them to determine the severity of the bone loss in the periodontally compromised incisor teeth. All examiners were calibrated for annotation and categorization of the severity of the bone loss. All the periapical radiographs for which the diagnosis of the 3 examiners did not agree were excluded. We also categorized the severity of bone loss based on the traditional classification by the International Workshop for Classification of Periodontal Diseases and Conditions (1999) in which the root is divided into three parts from the CEJ to the root apex. The first part represented the coronal third, the second the middle third, and the third the apical third. The severity of the bone loss has been defined as mild if the bone loss is in the coronal third of the root, moderate when in the middle third, advanced when in the apical third of the root length [17], and healthy when no vertical or horizontal alveolar bone loss was present.

Using a combination of transfer learning models with CNN architecture, the radiographic images were used to determine the optimal CNN algorithm in Python. The data set was divided randomly into 70% training dataset, 20% validation dataset, and 10% testing dataset. The image was exported manually in high-quality “PNG” format and examined and manually cropped to show only the tooth boundaries. The images were classified in binary (healthy or disease) and multiclassification (normal, mild, moderate, severe). Each image was resized to equal size of 150 × 150 pixels. The pixel value was normalized to a value between zero and one, Greyscale.

This study was designed to use a CNN-based model called VGG-16 (Visual Geometry Group) network architecture with the TensorFlow and Keras libraries. This architecture is the most popular and effective deep learning model for image classification problems [28]. The use of previous knowledge in rebuilding machine learning for the collected dataset is better than starting from scratch to solve a new image classification proble [24]. Transfer learning well avoids building deep convolution networks to local optimal and over-fitting problems [26].

**Fig. 1:**
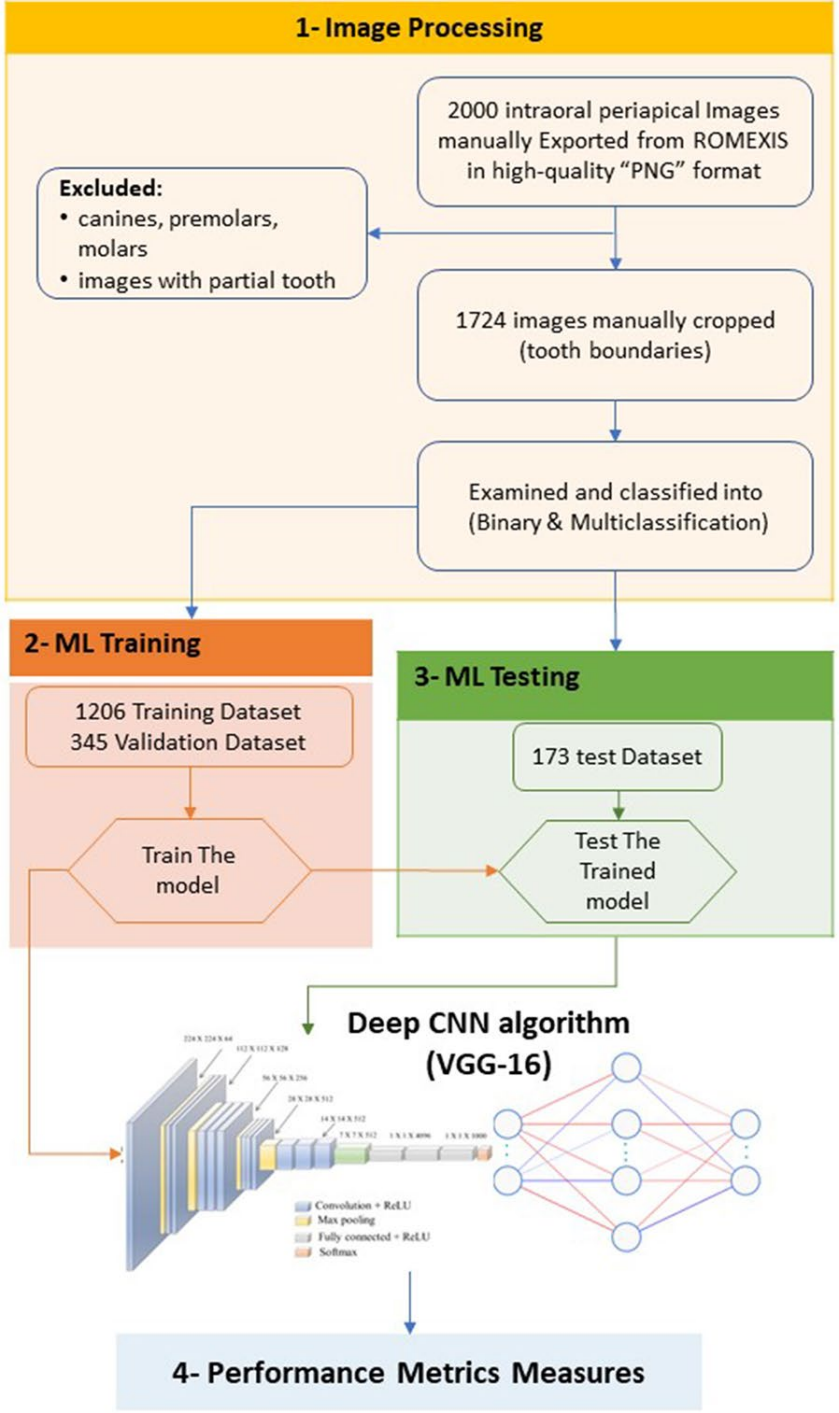
Scheme of the general process in the methodology

We used the transfer learning approach to classify our dataset. Transfer learning is a process of sharing one domain's knowledge with another domain. The proposed model consists of 13 convolutional layers and 2 dense layers because we fine-tuned the network. The dense layer contained 256 neurons, the last layer 4 neurons for the multi-classification, and 2 neurons for the binary classification. Also, in the drop layer to prevent overfitting, we randomly dropped 50% of the neurons. We used the RMSprop optimizer with the loss of categorical cross-entropy for model learning. We trained the model using 100 epochs and 16 batch sizes. Obtaining the result in a short time with highly accurate prediction and diagnosis is an essential factor in periodontal treatment. There is ongoing research to improve the accuracy and the speed of radiology AI [23]. The entire process of the methodology is presented in Fig. 1.

### Statistical analysis

The dental radiographic image dataset was evaluated for two different classifications, the binary classification (healthy or disease) and the multi-classification (normal bone, mild bone loss, moderate bone loss, and severe bone loss). For the binary classification, the images of the dataset was divided in a training dataset (n = 1206; 70%), a validation dataset (n = 345; 20%), and a test dataset (n = 173; 10%), and for the multi-classification the training dataset (n = 1206; 70%), validation dataset (n = 345; 20%), and test dataset (n = 173; 10%). The training dataset was used by the CNN model to learn the RBL detection and distinguish between the normal and abnormal periodontal bone levels in both types of classification.

The validation dataset was used to analyze the CNN performance and generate the best weights for a deep CNN algorithm model. Finally, the test dataset was used to evaluate the CNN prediction models by applying a confusion matrix, accuracy, precision, recall, F1-score, Matthews Correlation Coefficient (MCC), and Cohen Kappa. The κ values for the Cohen kappa were classified as follows: 0, poor; 0.00–0.20, weak; 0.21–0.40, fair; 0.41–0.60, moderate; 0.61–0.80, substantial; and 0.81–1.00, almost perfect agreement.

## Results

### Demographic data of the patients

The dataset of the dental radiographic images consisted of a total of 1724 periapical radiographic images. Almost half (n = 814, 47.21%) were classified as normal/healthy teeth and 910 (52.78%) as non-normal/deceased teeth with alveolar bone loss based on the binary classification. Of the non-normal teeth, a multiclass classification was performed with 511 (29.64%) categorized as mild, 290 (16.82%) as moderate, and 109 (6.32%) with severe bone loss.

### Model performance result for PCT classification

#### Confusion matrices and Accuracy

**Fig. 2:**
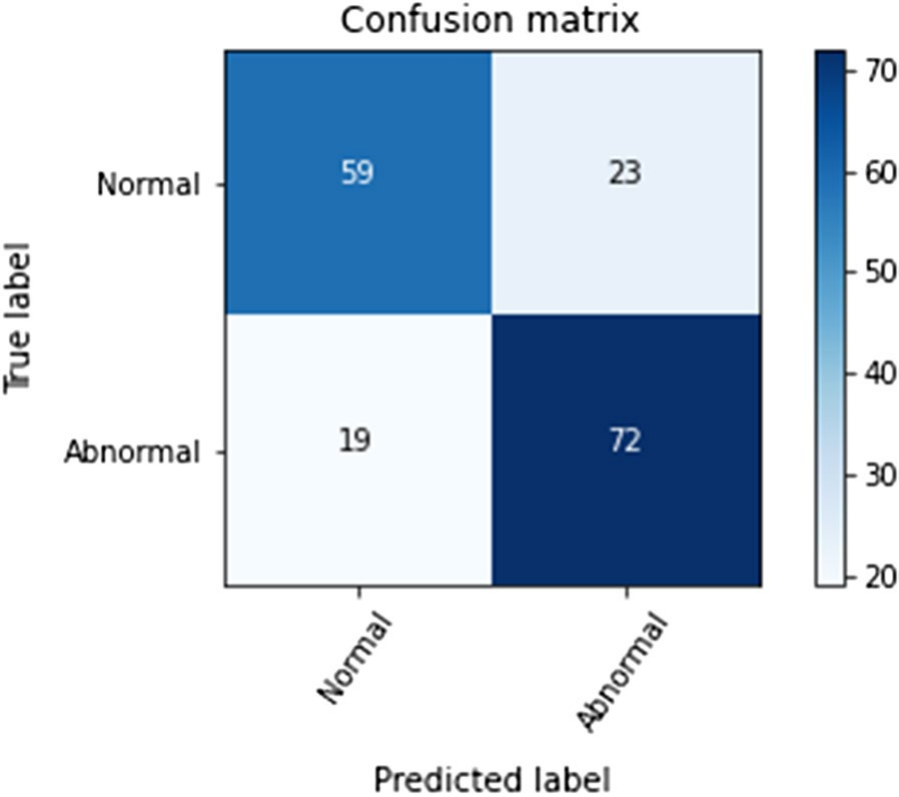
Binary class confusion matrix using a deep CNN classifier

**Fig. 3:**
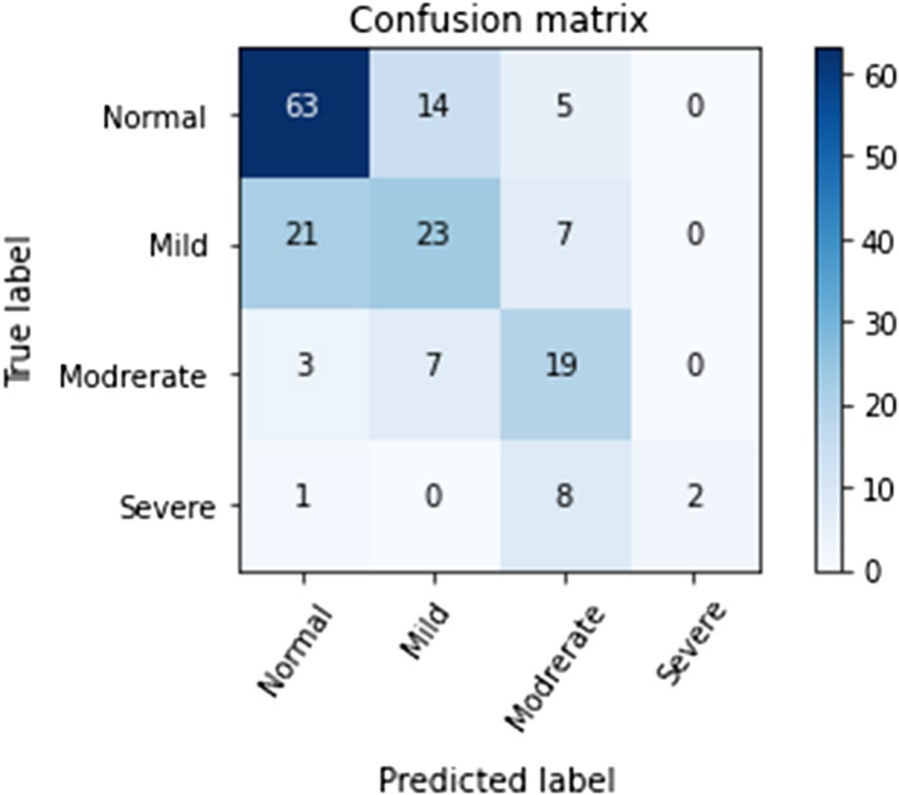
Multi-classification confusion matrix using a deep CNN classifier

The results of the confusion matrices for the alveolar bone levels with and without normalization using the CNN classification when testing the model training and testing it for the binary classification and misclassification are shown in Figs. 2 and 3. The color gamut of shade varies and gets darker according to the proportion of the correct value with the classification. The diagonal components are the number of images that were predicted correctly, and the label of prediction matches the actual true label. However, the non-diagonal components were misjudged by the classifier. The deep CNN had an accuracy of 73.04% and 59.42%, for the binary and multi-classification, respectively. There was a significant difference in the accuracy result of predictive between both binary and multi-classification approaches (*p* = 0.037).

The total diagnostic accuracy of the alveolar bone levels in the binary classification was 73.04% and the highest diagnostic accuracy was for the presence of alveolar bone loss (72%), and the lowest the absence of alveolar bone loss (59%). The total diagnostic accuracy for the multi-classification was 59.42%, with the highest diagnostic accuracy for the presence of normal alveolar bone, followed by mild bone loss, moderate severity, and the lowest severe alveolar bone loss.

#### Precision, recall, and F1-scores

The ML classification performance and model prediction efficiency and effectiveness were evaluated using accuracy, precision, recall, F1-score (Tables 1 and 2). The precision, recall, and the F1-scores for the binary classifier were above 70%, indicating that ML is a good classifier for the presence and absence of alveolar bone. In our findings, a score of 0.75 indicates a good model ability in predicting the correct class for the presence and absence of alveolar bone. The precision ranged from 45 to 83% for the multi-classification. The recall and F1 scores ranged from 45 and 70%. The mild bone loss had the lowest sensitivity as well as the lowest F1 score (0.45). The normal alveolar bone levels had an F1 score of 0.70, the highest across all the stages. However, the values differed significantly for the multi-classification indicating that it may only fairly classify the severity of the bone loss (Table 2).

**Table 1:** Experimental results for the transfer learning model

Class	Correct validation	Accuracy of validation (%)	Correct test	Accuracy of test (%)
*Experimental results per each class of the learning model – multi-classification*				
Normal	114	69.93	63	76.82
Mild	46	45.09	23	45.09
Moderate	35	60.34	19	65.51
Severe	10	45.45	2	18.18
*Experimental results per each class of the learning model – binary classification*				
Normal	140	85.88	59	71.95
Abnormal	112	61.53	72	79.12

**Table 2:** Statistical evaluation of the learning model

Class	Precision	Recall	F1-score	Support
*Multi-classification statistical evaluation*				
Mild	0.45	0.45	0.45	102
Moderate	0.52	0.60	0.56	58
Normal	0.70	0.70	0.70	163
Severe	0.83	0.45	0.59	22
Macro avg	0.63	0.55	0.57	345
Weighted avg	0.60	0.59	0.59	345
*Binary classification statistical evaluation*				
Abnormal	0.73	0.77	0.75	182
Normal	0.73	0.69	0.71	163
Macro avg	0.73	0.73	0.73	345
Weighted avg	0.73	0.73	0.73	345

#### Matthews correlation coefficient (MCC)

Due to the imbalance in the number of images in each category of the bone level loss dataset, the MCC index was used to evaluate both models’ performance. A correlation of 0.51 and 0.65 for the binary and multi-classification signifies the that predicted class and the true class is moderately correlated.

#### Cohen Kappa

The assignment of the presence/absence of bone loss between the ML and the periodontists showed moderate agreement (k = 0.512). However, the agreement for multi-classification was fair (k = 0.41).

#### Sensitivity and specificity

The detection of healthy versus diseased alveolar bone using machine learning as a diagnostic marker had a sensitivity of 73% and a specificity of 79.1%, with the detection of bone loss by the periodontist as the gold standard.

## Discussion

In this study, we evaluated the diagnostic performance of a CNN-based model VGG-16 to detect periodontal bone loss and classify the alveolar bone levels in teeth affected by periodontal disease. The presence of alveolar bone loss was detected with high accuracy by the system. Our findings revealed that deep CNN had a diagnostic accuracy of 73.04% in detecting an alveolar bone loss in the anterior teeth of both arches. The total diagnostic accuracy for the multi-classification was 59.42%, with the highest diagnostic accuracy for the presence of normal alveolar bone, followed by mild, moderate, and severe alveolar bone loss.

Our findings are supported by Lee et al. [15] who developed a deep learning model to classify periodontally compromised posterior teeth from periapical radiographs. They revealed a diagnostic accuracy of 81% for periodontally compromised premolar teeth and 76.7% for molars, similar to our findings of 73% for the anterior teeth. Another study by Lee Chun demonstrated a diagnostic accuracy of 0.85 and no significant difference in the RBL percentage measurements determined by the DL and examiners and high sensitivity, specificity, and accuracy for the different stages, all over 0.8 [14]. Other models used DL to detect RBL or calculate the RBL percentage from the panoramic radiographs and to assign periodontitis staging from the panoramic radiographs [7–16]. Although these models on the panoramic radiographs had good accuracy and reliability in assessing the bone level on the panoramic radiographs, it is generally not recommended to rely on panoramic radiographs due to the presence of distorted images, overlapping objects, and low resolution [1–18, 21].

The proposed model is the first model that assigned a severity category based on the traditional classification by the International Workshop for Classification of Periodontal Diseases and Conditions (1999) with the severity of the bone loss has been characterized as mild with bone loss is in the coronal third of the root, moderate with bone loss is in the middle third of the root and advanced when in the apical third of the root length [17]. Healthy indicated no vertical or horizontal alveolar bone loss.

The current study was based on periapical radiographs, the standard radiographic images for periodontal diagnosis. A major highlight of our study is that we have not excluded caries, restorations, and endodontically treated teeth to increase the complexity and simulate real-life scenarios as much as possible. The current results confirmed the CNN model, designed to detect the presence and absence of RBL and categorize the bone loss based on the severity, may assist in saving time and produce confirmatory decisions for RBL detection and categorization of the severity. The implication is that the clinicians would not have to assign a bone loss stage by manually calculating the RBL percentage for each tooth, a very time-consuming process.

The deep CNN algorithm yielded promising results in various fields of medicine, including imaging [4–15]. In our study, we performed supervised deep learning using a CNN-based model called VGG-16 (Visual Geometry Group) on a periapical radiographic dental dataset. We confirmed that the results had a close predictive accuracy compared with the periodontists. The deep CNN algorithm had a higher diagnostic accuracy to distinguish between no bone loss from bone loss in the upper and lower incisor teeth and the deep CNN algorithm was better trained and optimized for the detection of a normal PCT. However, the accuracy of categorizing the severity was lower. The diagnostic accuracy for severe bone loss was the lowest overall, and the trained deep CNN algorithm was poorly optimized for the detection of severe bone loss. Further studies on the mechanisms underlying deep CNN algorithms are necessary. The sensitivity, specificity, and the F-measure demonstrated acceptable performance for the binary classification, which supports the appropriateness of this method. Other topics for future research include additional techniques for improving the system output, such as applying more advanced augmentation techniques, extending the dataset, and using more recent CNN architectures.

In summary, this study found that a faster R-CNN trained on a limited number of labeled imaging data had a good capability of detecting the presence of bone loss and a satisfactory detection ability for the severity of bone loss in teeth. The application of a faster R-CNN to assist in detecting bone loss may reduce diagnostic effort by saving assessment time and automating screening documentation. A good quality tooth edge is important if the periodontal tissue is damaged in order to increase the performance accuracy of a PCT model, both diagnostically and predicatively. The Deep CNN algorithm can automatically extract features from PCT images and identify diverse characteristics in the input image, such as spots, corners, edges, or progressively complex characteristics, including patterns, structures, and shapes [15]. The VGG-16 has a powerful advantage in deciphering the detection problem, supporting its use in the present study [24].

The strengths of our study include the fact the consideration of imbalanced data, for which accuracy may not the best measure of the performance of a classifier. We considered other measures such as precision and recall (also known as sensitivity) as the most appropriate measure for the performance of imbalanced data.

## Limitations

A limitation of this study is that the machine was only tested to detect and categorize alveolar bone loss, not to diagnose periodontitis. It is noteworthy that only 2-dimensional periapical radiographs are insufficient for a complete diagnosis or prediction of PD. In order to make a more accurate diagnosis and prediction of PD, radiographic and clinical data must be reviewed comprehensively, including the patient's history and a comprehensive periodontal examination. Deep CNN algorithms based on periapical radiographic images alone do not provide sufficient evidence to diagnose and predict periodontally compromised teeth, but they may be useful as a reference.

We had a heterogeneous distribution of images, based on the severity with less severe and moderate images. This may have contributed to the reduction of the diagnostic accuracy for the multi-classification. To develop an advanced deep learning algorithm with the upgraded performance it is important to consider the design of the algorithm and the use of a balanced training dataset with high quality. To overcome this limitation of imbalanced image classes required for deep learning, we collected only high-quality images that were classified by the periodontist. Learning transfer and preprocessing techniques, including image augmentation and enhancement, were used to avoid overfitting and to normalize the model [15].

The performance of the machine learning algorithms changes with an increasing data size [27]. Additional research on a larger dental image dataset with an equal distribution of the groups and deep CNN algorithms for classification is required.

The images were cropped and resized to 150X150 pixels due to practical constraints. Another limitation of our study was that we performed our results on Google Collab with limited memory space, however, memory limits are exceeded if the input dimensions of images are increased (the memory size of google Collab is 12 GB). To process 1800 images with limited resources, Google Collab was used. We downgraded the input dimension to 150 to utilize the affected resource. Furthermore, we tried 224 dimensions with a reduced number of images sample to see if the utilization of memory was affected, but the results were not good. The accuracy was lower than what we have now, and we recommend investigating the impact of the periapical dimension on deep learning performance.

Understanding the difference in human dentition is crucial in studies. On a superficial level, the teeth of different individuals may appear similar but on closer examination, they reveal significant variation in both size and shape [3]. This variation in the image dataset for upper and lower teeth might affect the model result. Maintaining a high quality that covers this variation within each class is important and studies are required to reduce the impact of this factor on the performance of the deep learning prediction and diagnoses.

Clinical trials comparing the identification of periodontally compromised teeth using conventional clinical and radiographic findings with and without the support of the CNN should be done. Because the differential diagnosis between healthy teeth and incipient PCT was made using only periapical radiographs, this study did not diagnose or distinguish between healthy teeth and incipient PCT.

## Conclusion

A deep CNN algorithm (VGG-16) was found to be useful to detect alveolar bone loss in periapical radiographs, as well as to detect the severity of bone loss in teeth. The results indicate that machines can perform better based on the level classification and captured characteristics of the image diagnosis. By optimizing the periodontal dataset, a computer-aided detection system should be able to aid in the detection and staging of periodontitis.

## Data Availability

The datasets generated and/or analyzed during the current study are not publicly available due [raw data generated from college of dentistry, KSAU-HS] but are available from the corresponding author on reasonable request.

## Abbreviations

CNN: Convolutional neural network
MCC: Matthews Correlation Coefficient
PCT: PCeriodontally compromised teeth
AI: Artificial intelligence
PD: Periodontitis
RBL: Radiographic bone loss
